# Intraspecific Differences in the Venom of *Crotalus durissus cumanensis* from Colombia

**DOI:** 10.3390/toxins14080532

**Published:** 2022-08-02

**Authors:** Ariadna Rodríguez-Vargas, Nohora Vega, Edgar Reyes-Montaño, Gerardo Corzo, Edgar Neri-Castro, Herlinda Clement, Francisco Ruiz-Gómez

**Affiliations:** 1 Grupo de Investigación en Proteínas, Universidad Nacional de Colombia, Bogotá 11001, Colombia; navegac@unal.edu.co (N.V.); eareyesm@unal.edu.co (E.R.-M.); 2 Departamento de Medicina Molecular y Bioprocesos, Instituto de Biotecnología, Universidad Nacional Autónoma de México, Cuernavaca 62210, Mexico; gerardo.corzo@ibt.unam.mx (G.C.); edgar.neri@ibt.unam.mx (E.N.-C.); linda@ibt.unam.mx (H.C.); 3 Grupo de Investigación en Animales Ponzoñosos y sus Venenos, Instituto Nacional de Salud, Bogotá 111321, Colombia; fruiz@ins.gov.co

**Keywords:** *Crotalus durissus cumanensis*, venom, antivenom

## Abstract

Biochemical and biological differences in the venom of *Crotalus durissus cumanensis* from three ecoregions of Colombia were evaluated. Rattlesnakes were collected from the geographic areas of Magdalena Medio (MM), Caribe (CA) and Orinoquía (OR). All three regionally distributed venoms contain proteases, PLA_2_s and the basic subunit of crotoxin. However, only crotamine was detected in the CA venom. The highest lethality, coagulant, phospholipase A_2_ and hyaluronidase activities were found in the MM venom. Also, some differences, observed by western blot and immunoaffinity, were found in all three venoms when using commercial antivenoms. Furthermore, all three eco-regional venoms showed intraspecific variability, considering the differences in the abundance and intensity of their components, in addition to the activity and response to commercial antivenoms.

## 1. Introduction

*Crotalus* genus belongs to the Viperidae family. It is geographically distributed from Canada to northern Argentina. The species *Crotalus durissus* (Linnaeus, 1758) has the widest range of geographical distribution, which is related to the ten subspecies that are described for the group [1]. Rattlesnakes inhabit savannahs, scattered forests and thickets, located in mountainous ecosystems of America [2]. *Crotalus durissus cumanensis* is a subspecies located in Colombia towards the Caribbean, Eastern Plains and Magdalena Medio regions [3,4] and belongs to the large *Crotalus durissus* complex that also includes *Crotalus durissus terrificus*, with a wide distribution in Brazil [5].

Although, neurotoxic activity of phospholipases type A_2_ (PLA_2_) and low molecular weight myotoxins prevail in the venom of rattlesnakes, in addition to hemocytotoxic effects related to proteases, Central and North American rattlesnake venoms contain proteolytic, hemotoxic, and edematogenic components, but there are few species with neurotoxic activity in their venoms [6,7,8,9]. In South America, venoms of rattlesnakes belonging to the *C. durissus* complex have components such as gyroxin, crotoxin, convulxin and crotamine, which give them additional neurotoxic activity [10]. *Crotalus durissus cumanensis* venom presents different pathophysiological activities, because it causes myotoxicity and coagulopathies besides neurotoxic effects [11,12].

Snakebite accidents caused by *Crotalus* sp. are considered of medical importance since they can seriously compromise the life of a patient. Studies of crotalid accidents in South America describe envenoming due to the presence of hematuria, myalgia, neurotoxic facies, and blood coagulation disturbances [13]. Also, indirect nephrotoxic effects are attributed to the venom due to muscle damage, procoagulant activity, and even intravascular hemolysis [11,14].

Between 2019 and 2021, the Colombian Epidemiological Surveillance System recorded an average of 5079 cases of snakebites, led by bothropic (60%) and then by crotalic accidents, the latter with an average of 3.5%. The accidents due to vipers, generally registered severe cases in 8% of the events [15,16,17].

Antivenom therapy is the only specific treatment approved for managing snakebite accidents [18,19], due to the efficacy in terms of neutralizing the toxic components of the venom and the effectiveness, referable to the reversal of the clinical manifestations of envenomation [20]. In Colombia, one of the available commercial antivenoms is produced by the Instituto Nacional de Salud (INS), which is a polyvalent heterologous serum, made by the inoculation of horses with the venom of *Crotalus* and *Bothrops* species. This antivenom is a mixture of complete immunoglobulins G precipitated with ammonium sulfate from equine hyperimmune plasma, which neutralizes *Bothrops* sp. *sensu lato* and *Crotalus* sp. *sensu lato* venoms, and by cross-reactivity, it is able to neutralize *Lachesis muta* and *Lachesis acrochorda* venoms [21]. In contrast, a foreign commercial antivenom, distributed in Colombia, from Instituto Bioclón of México, is a polyvalent antiviperid (Antivipmyn-Tri^®^) digested with pepsin (fabotherapeutic F(ab’)_2_ type), from the plasma of horses hyperimmunized with a venom mixture of *B. asper* from Mexico, *C. durissus* from South America and *Lachesis muta* from Central America [22,23]. Concerning the neutralization capacity of the two commercial antivenoms, a volume of 10 mL of the INS antivenom neutralizes 10 mg of *Crotalus* sp. venom [21], and 10 mL of Antivipmyn-Tri^®^ neutralizes 15 mg of *Crotalus* sp. venom [23,24].

Concerning the intraspecific variability in rattlesnake venoms, it has been determined that the venom of *C. d. cumanensis* of the species found in Venezuela, presents individual variability regarding fibrinolytic, hemorrhagic and lethal activity [25,26]. Snakebite accidents with the same species presents particular signs of neurotoxicity [27], according to its toxinological characterization [28]. Similarly, for the species *C. d. cumanensis* from Colombia, its ontogenic variation [29,30], proteomics and response to antivenoms [31,32] seems different from the Venezuelan species. PLA_2_s and proteases from *C. d. cumanensis* venoms from Colombia have been studied [33,34]. Additionally, their cytotoxic, antiplasmodial, myotoxic and anticoagulant effects have been reported [35,36,37].

Considering that the main distribution of *C. d. cumanensis* species is widespread in Colombia, and that venoms have differences in their composition according to geographic location, ontogenetic variability, diet and sex, and by evolutionary strategy in response to the ecological pressure of the predator vs. prey relationship [38,39], a biochemical study of ecoregional venoms of *C. d. cumanensis* was carried out using representative pools of venoms from Magdalena Medio (MM), Caribe (CA) and Orinoquía (OR).

## 2. Results and Discussion

### 2.1. Phospholipases A_2_ and Low Molecular Weight Myotoxins as Hallmarks in Protein Profiles

**Figure 1:**
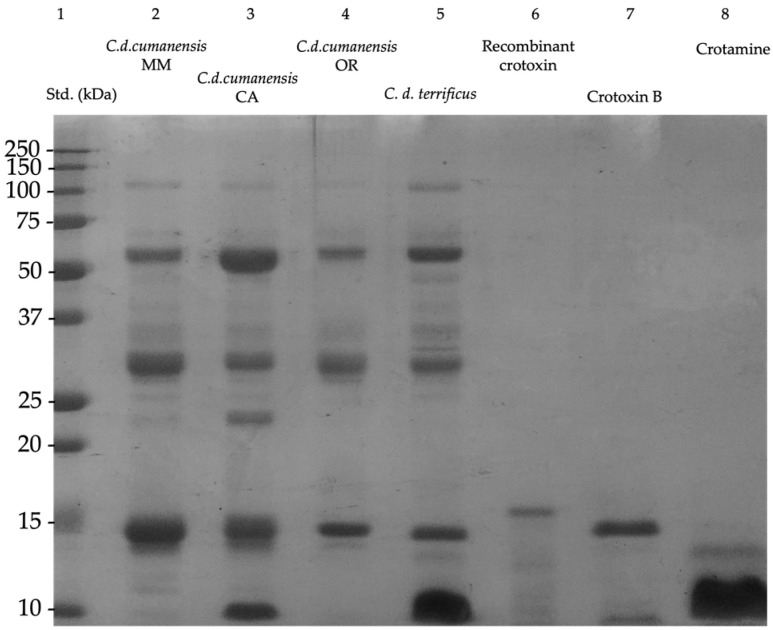
SDS-PAGE 12.5% under reducing conditions of *Crotalus durissus cumanensis* venom and controls. 20 μg of protein were seeded for recombinant crotoxin B [40] and native crotoxin B; for the other samples 10 μg of protein were seeded. Std.: molecular weight standard; MM: Magdalena Medio; CA: Caribe and OR: Orinoquía region.

PLA_2_s, proteases and low molecular-weight components are common in rattlesnake venoms. Figure 1 shows an SDS-PAGE comparing the ecoregional venoms with apparent molecular weights from 10 to 110 kDa. Metalloproteases (SVMP) and serineproteases (SVSP) are usually in the range from 20 to 70 kDa, with some SVMP even reaching the 110 kDa. Bands around 14 kDa correspond to PLA_2_s, mainly represented by the basic subunit of crotoxin (Figure 1, lanes 6 and 7, recombinant crotoxin was only used for visual comparison). The presence of a thick band ≤ 10 kDa, related to crotamine, was only observed in the CA venom, and it corresponds to the same band (myotoxin) in *C. d. terrificus* venom used as referential control.

Analysis of the relative intensities of the bands obtained by SDS-PAGE are shown in Table 1. Bands of high, medium, and low molecular weight, as well as peptides, were grouped, and the sum in each group was assumed as 100%. Thus, for example, the relative intensity of each band within the group of peptides (molecular weights ranging from 8 to 10 kDa) was obtained by comparing it with their respective 100%. In general, viperid venoms present LAAO and some SVMP that appear in molecular weights greater than 50 kDa. CA venom accounted for 44% of this group. In the range between 25 and 50 kDa, 47% were described for MM, while OR only presented 16%; this group has protein families such as SVMP, SVSP and cysteine-rich secretory proteins (CRISP). The most important group for these venoms was PLA_2_, for which greater intensity was observed in CA, followed by MM (43% and 39%, respectively). Likewise, CA presented higher intensity for bands of lower molecular weight, where crotamine stands out, being 55% of the intensity analysis [38,41].

**Figure 2:**
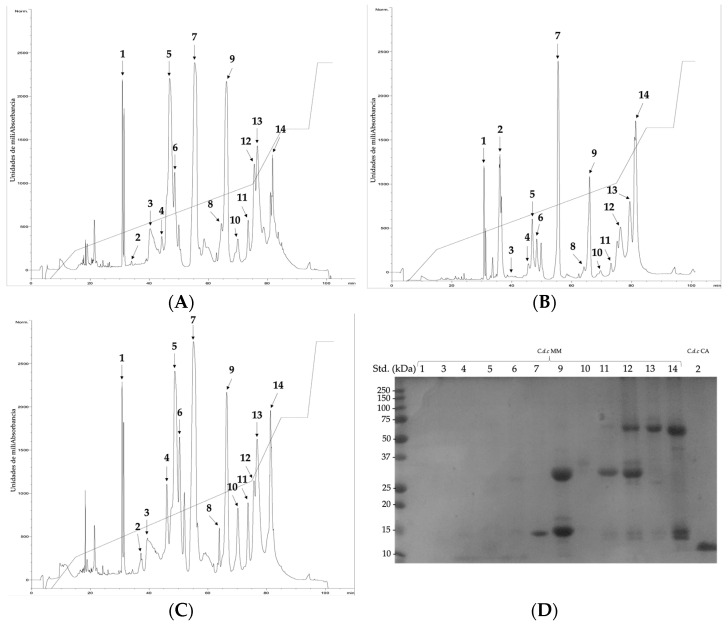
RP-HPLC chromatographic elution profiles of *C. d. cumanensis* venom from (**A**) Magdalena Medio (MM), (**B**) Caribe (CA) and (**C**) Orinoquía (OR); (**D**) SDS-PAGE 12.5% under reducing conditions of MM and CA fractions obtained by RP-HPLC. 5 μg of total protein from each venom were seeded. Std.: molecular weight standard.

By RP-HPLC, fourteen coincident abundant fractions were determined among the three venoms (Figure 2). Fraction 3 on MM venom had a molecular mass of 1239.1 Da and fractions 7, 9, and 11 had a molecular mass of 14,395.6 Da, 15,424.5 Da, and 14,439.0 Da, respectively, (see also Table 2). These last three fractions are related to molecular masses of phospholipases A_2_ [59,61]. By SDS PAGE, fraction 7 (Figure 2D) matches the basic subunit of crotoxin (or crotoxin B), as described [62]. Fraction 8 was found in the OR venom (Figure 2C and Table 2), which eluted at 64.1 min and showed a molecular mass of 13,550.0 Da, also related to PLA_2_.

It had already been shown for a venom pool of Colombian *C. d. cumanensis* that several fractions in the same range had molecular masses and peptide sequences related to the subunit B of crotoxin; therefore, it is highly possible that the four fractions, with similar molecular masses, correspond to isoforms of the same protein [10,31].

Fraction 3, purified from the MM venom by RP-HPLC (Figure 2A), had a small molecular mass, which may relate it to vasoactive peptides, for example, bradykinin-inhibitory or potentiating peptides [63,64,65,66].

Regarding CA venom, fraction 2 was important, eluted at 36.6 min (Figure 2B), whose electrophoresis band was found at ≤10 kDa, with a reported molecular mass of 4910.9 Da (Table 2), corresponding with crotamine. For the three species studied, differences in crotamine content stand out. For venom CA represented ~36% by SDS-PAGE, for MM 15%, and for OR it was not found.

Crotamine is a low molecular-weight myotoxin, characterized in South American rattlesnake venoms [67], as well as different North American species, *C. molossus nigrescens*, *C. s. scutulatus*, *C. oreganus*, *C. tzabcan*, *C. culminatus*, among others. It has an average molecular mass of 5 kDa [58,68] and has a net positive charge [58]. Since the two essential neurotoxic components are easily identified in CA venom, the relative abundance was calculated according to that obtained by RP-HPLC. Thus, crotamine and crotoxin B subunit together represent ~29% of the total protein composition in the venom, which is related to neurotoxic manifestations during envenomings. For Venezuelan *C. d. cumanensis* the relative abundance of crotamine was 13% [12], while for other species of *Crotalus* from South America, it fluctuates between 5 and 29% [67]; with the exception of the Brazilian snakes, *C. ruruima* which has only 1.5% of crotamine [12], and *C. d. cascavella* that does not have crotamine in its venom. As far as is known, the decrease in crotamine concentration seems to be compensated by an increase in PLA_2_, as is tended to show in MM and OR venom in the present study [68].

When adding the abundances of fractions 7 to 11, which coincide with the presence of PLA_2_ [63], and which include the crotoxin B subunit, 44% is represented by MM. This is consistent with the intraspecies variation that supports the theory of *C. d. cumanensis* as an evolutionary intermediary whose venom has the hemotoxic characteristics of the northern *Crotalus* species and the neurotoxic characteristics of those of southern America [12].

As proposed, the neurotoxicity associated with increased expression of crotoxin and crotamine represents an adaptive trend related to the diversification of *Crotalus durissus*. The same authors found that *C. d. terrificus* (Brazil), *C. ruruima* (Brazil), *C. d. durissus* (French Guiana) and *C. d. cumanensis* (Venezuela) had crotamine in their venoms, while *Crotalus simus* (Costa Rica) did not [12].

### 2.2. Heterogeneous Enzyme Activity and Related Cytotoxicity

**Figure 3:**
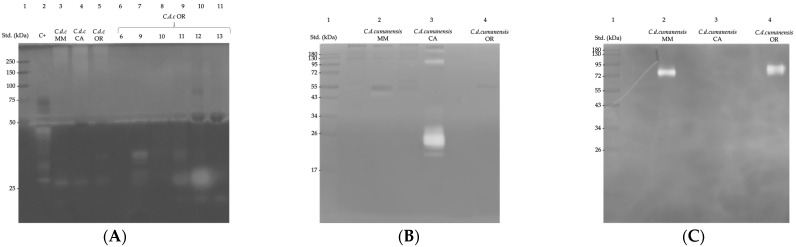
Protease activity zymogram in 12.5% gels for *C. d. cumanensis* (*C.d.c.*) venoms using (**A**) gelatin and (**B**) casein as substrates. *Bothrops ammodytoides* venom was used as a positive control (C+). In (**C**) the hyaluronidase activity zymogram for the three *C. d. c.* using 10% hyaluronic acid substrate in 10% polyacrylamide gel. 5 μg of sample were seeded. Std.: molecular weight standard.

On the gelatin substrate, all venoms showed protease activity towards 250 kDa and, most notably, at 27 kDa, most prominent in MM (Figure 3A, lanes 3–5). Fractions 9, 11 and 12 of OR showed great activity on 27 kDa. OR venom exhibited a faint band around 35 kDa, which was more evident in isolated fractions 9, 10 and 11, being more noticeable for fraction 9. Intense bands were observed, close to 20 kDa for fractions 11–13. Compared to the positive control, the activity was different since no bands appeared between 35 and 50 kDa for the whole venoms and the fractions. Metalloproteases are a diverse group of multidomain proteins. Class P-III proteins have already been described in several *Crotalus* sp. with an estimated molecular weight between 60 and 100 kDa [42,59,69]. Those refer to the venom of *C. d. cumanensis* could belong mainly to this group [31]. During envenomation, these enzymes damage the basement membrane of blood capillary vessels. As a result, fibrinogenolysis, platelet aggregation, and hemorrhage occur [42,43,44]. This activity could be more evident when the substrate was gelatin, a polymer obtained from the hydrolysis of collagen, one of the fundamental components of the extracellular matrix. With the casein substrate, protease activity was important for CA venom, with intense well-defined bands >26, 95 and 180 kDa. For the MM venom, a very faint band was observed near 50 kDa (Figure 3B).

Serineproteases, which can represent ~6% of the proteome of Colombian *C. d. cumanensis* [31], are enzymes with molecular weights ranging between 26 and 37 kDa [48,49]. They act over components of the blood coagulation cascade, increase fibrinolysis, and cause disturbances on platelet aggregation and on the kallikrein-kinin system [48,50]. Thrombin-like serineproteases (TL-SVSPs) are an important subgroup due to their fibrinolytic effect. This activity could be seen on the gel polymerized with casein; however, the effect of SVMP is also reflected on the same substrate.

Zymograms for the venoms of *C. aquilus*, *C. polystictus* and *C. molossus nigrescens*, on gelatin and casein substrates, showed wider ranges of protease activity, occupying the range between 25 kDa and close to 200 kDa [41].

Hyaluronidase activity was detected in proteins of ~80 kDa only for MM and OR venoms (Figure 3C). HYAs from snake venoms have molecular weights ranging from 59 to 115 kDa due to structural heterogeneity related to post-translational modification, usually N-glycosylation [45,46]; truncation of transcripts or deficient characterization [70]. They are present in scarce amounts in venoms; therefore, their activity is apparently low as seen in the respective zymogram, although they are of great importance, since they allow the entry of other components of the venom to generate greater toxicity [71]. For *C. d. terrificus*, there is already an identified HYA whose molecular weight is approximately 64.5 kDa [41,45]. In some assays, *Crotalus atrox* has also shown hyauronidase activity close to 68 kDa [38]. CA venom has a deficient hyaluronidase activity; it could be suspected that it has a compensation given by crotamine. Although it does not have the same effect as a dispersion factor, because of its low molecular weight, it has an easy-to-reach myotoxic/neurotoxic effect by mechanisms attributed to its toxicodynamics.

**Figure 4:**
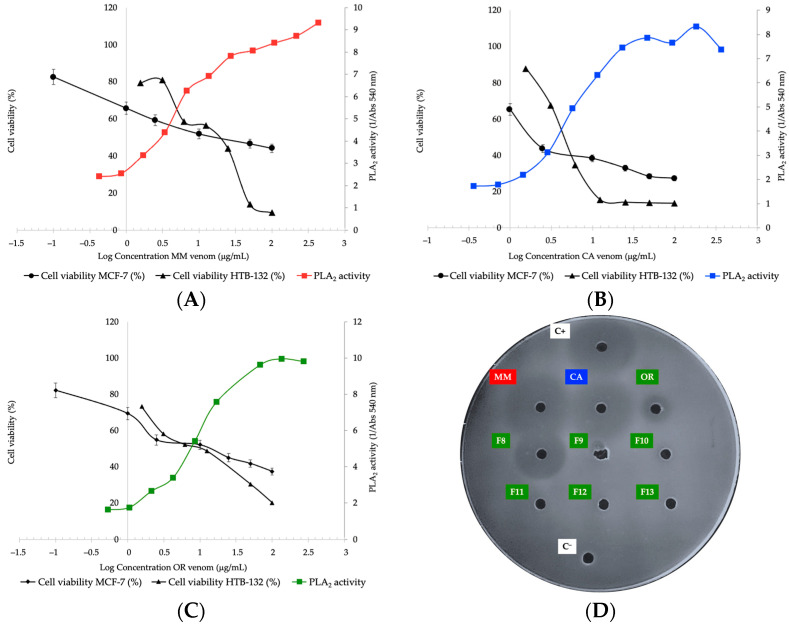
Determination of the PLA_2_ activity vs. cell viability in tumor lines MCF-7 y HTB-132 for the venom *C. d. cumanensis* from (**A**) Magdalena Medio (MM), (**B**) Caribe (CA) and (**C**) Orinoquía (OR). Qualitative PLA_2_ assay (**D**) showed the formation of translucent halos around each well. A 3 µg sample was seeded in each well. Reading performed at 24 h. *Bothrops ammodytoides* venom was the positive control (C+); milliQ water as negative control (C^−^); fractions (F) 8–13 of the OR venom, collected from RP-HPLC.

At venom concentrations of 437 μg/mL (log 2.6), PLA_2_ enzyme for MM is active with a tendency to increase, while for OR venom the activity is constant from 274 μg/mL (log 2.4), and likewise for CA at 186 μg/mL (log 2.3) (see Figure 4). Results are corroborated if comparing the formation of a translucent halo where MM had 86% of the positive control area, CA 78%, and OR 21%. The purified fraction 8 of OR venom represented 56% of the reference area, exceedingly almost three times that of its complete venom (Figure 4D). PLA_2_ activity appears with a clear hyperbolic trend for the three venoms. As the amount of enzyme increases, for CA and OR venoms, the curve reaches a saturation plateau. Although it is possible that this behavior is explained by the fixed amount of substrate used, the growth of the activity curve for the MM venom is remarkable, which coincides with the large halo of degradation of the substrate for its qualitative PLA_2_ activity.

On the MCF-7 and HTB-132 cell lines, the three venoms were cytotoxic, which suggested that PLA_2_ could be involved in this process, both due to relative abundance and activity [71]. Figure 4 shows a dose-response effect: higher amounts of total protein increase PLA_2_ activity and decrease cell viability. The IC_50_ values obtained for the MCF-7 cell line showed that a lower concentration of MM venom is required compared to OR and CA, which agrees with the content of PLA_2_, higher for MM and lower for CA. However, when comparing the venom concentrations in which there is PLA_2_ activity versus the cytotoxicity on MCF-7, results showed that the intercept exceeds, almost up to 18 times, the IC_50_ calculated for that cell line (i.e., MM venom) (Table 3).

MCF-7 comes from invasive ductal breast adenocarcinoma, isolated from the pleural cavity, has a luminal/epithelial subtype and is associated with a good medical prognosis, which is related to the low concentrations found for its IC_50_ [72]. Further studies for cytotoxicity on the MCF-7 line should be carried out to find a justification due to inner characteristics of the cell line or to some other components of the venoms that affect cell viability. Cytotoxic effect of the proteins in snake venoms is related to their structural nature and the target cells. Thus, the SVMP exert their proteolytic activity on the vascular endothelium and myocytes, in addition to the increase in inflammatory mediators that worsen it. PLA_2_ cause hydrolysis of membrane phospholipids on cells of the neuromuscular plate and red blood cells. Due to LAAO’s catalytic effect, cellular oxidative stress is induced leading to an apoptotic outcome; hyaluronan degradation products, typical of the extracellular matrix (ECM) of soft connective tissues, by HYAs can generate alterations in signal transduction, and C-type lectins through the recognition of carbohydrate side chains on the cell surface [47,73,74].

In addition to its myotoxic activity, crotamine has a cytotoxic and antiproliferative effect, being selective for cell lines with a high rate of cell division, such as tumor cells. Furthermore, its activity is due to an electrostatic interaction between negatively charged membrane components of cancer cells and positively charged crotamine residues, as well as the possibility of internalization by endocytosis and accumulation in lysosomes [58,75,76]. Finally, disintegrins recognize tumor cell-associated integrins and, through their interaction, appear to induce caspase-independent apoptosis [77,78].

However, the crossing of the curves of PLA_2_ activity vs. cytotoxic activity on the HTB-132 line does indicate a direct relationship between the two parameters, for the MM and CA venoms, because the value of the intercepts is very close to the IC_50_ value (Table 3). HTB-132 is defined by triple negative basal type cells of invasive ductal breast adenocarcinoma, with epithelial morphology, which is associated with a poor prognosis [79], but despite its aggressiveness, it used relatively low concentrations of venom to achieve cytotoxicity.

PLA_2_ from snake venoms are characterized by having a molecular weight in a range between 13 and 19 kDa [59]. They are calcium-dependent enzymes that hydrolyze the 2-acyl groups of 3-sn phosphoglycerides, with toxicological effects such as myonecrosis, cardiotoxicity, platelet aggregation/anti-aggregation, and pre and postsynaptic neurotoxicity [69]. PLA_2_ activity for a pool venom of *C. d. cumanensis* showed that a dose of 100 µg/mL, enzymatic activity did not reach saturation either. Cytotoxic activity was demonstrated on peripheral blood mononuclear cells with a dose of 18.23 ± 0.57 µg/mL, and on K562 cells, from chronic myeloid leukemia, with a dose of 2.34 ± 0.19 µg/mL, showing cell viability below 5% for the whole venom and purified subunit B of crotoxin, unlike other PLA_2_ isolated from the same venom [35]. PLA_2_ called Cdcum6, also purified from Colombian *C. d. cumanensis* venom, showed greater cytotoxicity on myotubes than on C2C12 skeletal muscle myoblasts, at a dose of 267 µg/mL [37].

### 2.3. Toxic Activity to Reinforce the Differentiation among the Three Venoms

MM venom showed more lethal test doses and better coagulating activity, compared to CA venom. Although OR venom did not have significant differences with MM in the LD_50_ assay, it did have differences with CA in the same test. The minimum defibrinating activity did not present statistically significant differences between the three venoms, so an average defibrination dose of 0.10 μg/g was obtained (Table 4).

Toxicological characterization of the Venezuelan *C. d. cumanensis* venom reported higher data than those presented here. In that case, the LD_50_ was 0.2 µg/g mouse, defibrinanting dose was 0.3 µg/g mouse, and the coagulant dose was 67.5 mg/L [25,28]. The three venoms of *C. d. cumanensis* seem to fit in phenotype II, which have low SVMP activity and high toxicity, since their LD_50_ is <1 µg/g mouse body weight [80]. Additionally (data not shown), a neurotoxic activity and lethality assay was performed with caudal intravenous application in mice of the ICR strain (CD1) weighing between 18 and 20 g. Separately, fraction 7 of OR venom, at a dose of 1.3 μg/g mouse, caused flaccid paralysis and subsequent death; and CA fraction 2, at a dose of 2 μg/g mouse, produced rigid paralysis and subsequent death. Compared to other rattlesnake venoms, the estimated LD_50_ for the venoms in this study is equal to the LD_50_ for *C. d. terrificus* and close to that of 0.1 µg/g mouse given for *C. simus* [81,82,83].

The lethal neurotoxicity coefficient (LNC) is a quantitative measure of the evolutionary pressure towards neurotoxicity gain and the lethal activity of the venom of *C. durissus* snakes that evolved after the invasion of South America from North America, and which is calculated on murine models. It is understood as the relationship between the average LD_50_ of each venom (in µg/kg) and the concentration of crotoxin plus crotamine in it (relative abundance) [12,63].

An estimate was made for the three venoms, with fraction 7 obtained by RP-HPLC, corresponding to subunit B of crotoxin and fraction 2 corresponding to crotamine. Thus, the LNC resulted in 3.8 for MM, 3.5 for CA and 4.3 for OR, being important to note that the maximum neurotoxicity is represented by CA venom. The LNC obtained are lower than those previously reported for the same species (27,4) but are still higher than that of *C. d. ruruima* and *C. d. terrificus*, which are references in the region (1.5 and 1.0, respectively) [12].

Defibrinating activity that turned out to be significantly better for MM venom is typically related to the presence of thrombin-like enzymes, whose molecular weight is between 26 and 33 kDa [49]. For *C.d. terrificus*, gyroxin has already been described as part of this group, with fibrinogenolytic activity [84].

Procoagulant effect, with equally lower doses for MM, has been described mainly due to the action of SVMP, which are toxins that act on different levels of the coagulation cascade, for example, activating factors V, X and prothrombin [85]. SVMP-III with known haemorrhagic, apoptotic and factor X activating effect could be related to this activity as mentioned above [69].

For other *C. d. cumanensis* venoms, LD_50_ ranges from 0.11–2.89 µg/g with defibrinating activity between 0.6 and 2.5 µg/g, while what was found in this study is below these values. However, for MCD, the range is markedly wider (17–157 mg/L) and our results fit within it [25,29,86].

### 2.4. Recognition of Complete Venoms and Some of Their Fractions by Antivenoms

Venom neutralization measurements were made using INS antivenom to calculate the median effective dose (ED_50_), which resulted in 1.4 mg of MM venom/mL antivenom (CL95% 1.01–1.62); 2.3 mg of CA venom/mL antivenom (CL95% 1.88–2.47), and; 0.6 mg of OR venom/mL antivenom (CL95% 0.57–0.75). The fact that more antivenom is required to neutralize CA venom may have to do with its low LNC and, therefore, its high toxicity. The values, however, turn out to be much lower than those reported in another study where the ED50 for INS antivenom, against a pool of *C. d. cumanensis* region OR, was obtained with values of 962 µL/mg venom [32].

**Figure 5:**
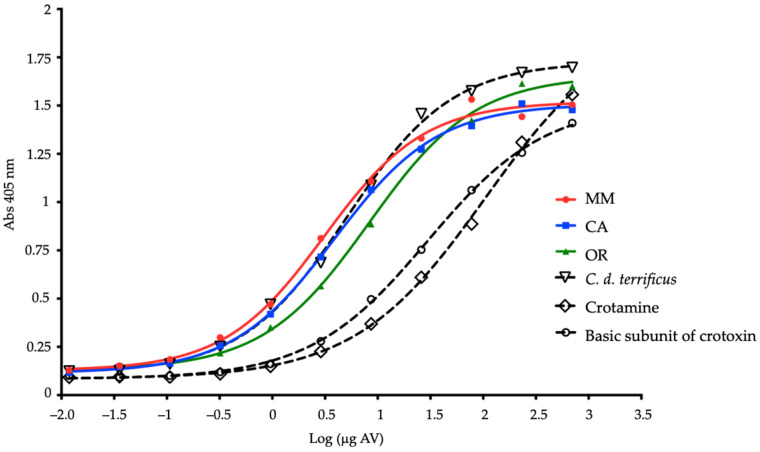
Instituto Nacional de Salud (INS) antivenom recognition against *C. d. cumanensis*, *C. d. terrificus*, crotoxin subunit B and crotamine, determined by ELISA. Each point represents the average of two measurements. MM: Magdalena Medio; CA: Caribe and OR: Orinoquía.

At a concentration of 4 µg/mL, venoms of each region were evaluated by ELISA to determine the recognition of INS antivenom, at two concentrations of 150 µg/mL and 700 µg/mL, the latter being the best (Figure 5). Recognition by the IgGs in the protein precipitate was observed. In addition, a linear trend was presented as the amount of antibody increased. The antigen-antibody (Ag-Ab) reaction is characterized by an equilibrium, where the lower and higher the dilution of the antibody, the reaction shifts towards the formation of a smaller amount of the Ag-Ab complex, observing that all venoms (including *C. d. terrificus*) showed concentration-dependent recognition, when compared to pre-immune serum. Crotamine requires a greater amount of antivenom for its recognition, in comparison with the B subunit of crotoxin and the rest of the samples.

Considering IC_50_ values in ELISA, defined as the concentration that provokes a response halfway between the maximum response and the maximum inhibited response, a good response was found towards the MM and CA venoms since it was 3.11 (log 0.49) and 3.85 (log 0.58), respectively. For OR venom, however, although recognition response was achieved, an IC_50_ higher than 8.0 (log 0.9) was obtained, while for the *C. d. terrificus* IC_50_ was reached with a value of 5.06 (log 0.7). For B subunit of crotoxin, and crotamine, since they are purified toxins, the IC_50_s are much higher: 24.6 and 64.3, respectively.

INS antivenom recognizes Colombian OR, MM, and CA venoms, such as *C. d. terrificus*, indicating that its components have common epitopes. It is important to highlight this cross recognition because some antivenoms do not adequately neutralize species of the *C. durissus* complex [12]. In the context of the dynamics of envenoming, crotoxin has a higher clearance due to its low molecular weight and rapid distribution towards its site of action [87]; however, in the ELISA test presented here, where there is preincubation antigen-antibody, INS antivenom demonstrated a satisfactory coverage of said toxin. INS antivenom exhibits immunoreactivity against *B. asper, B. atrox, L. acrochorda, B. schlegelii, B. punctatus* venoms, and, with lower levels of recognition, *C. d. cumanensis* [32].

**Figure 6:**
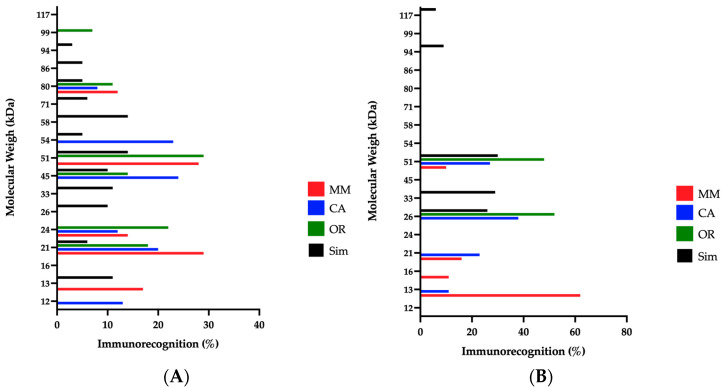
Western blot assay analysis of antivenoms against the venoms of *C. d cumanensis* and *Crotalus simus* (Sim) for the antivenom from (**A**) Instituto Nacional de Salud (INS, Colombia), and (**B**) Antivipmyn-Tri^®^ (Laboratorio Bioclon, Mexico). MM: Magdalena Medio; CA: Caribe and OR: Orinoquía.

In the Western blot analysis, densitometry was considered to account for the percentages of immunorecognition. Several bands of *C. d cumanensis* venoms, with molecular weights between 12 and 117 kDa, under non-reducing conditions, were recognized by INS antivenom. Low molecular-weight components were best recognized for MM by INS (17%) and Antivipmyn-Tri^®^ (73%) antivenoms (Figure 6A,B). For the same range, *Crotalus simus* venom (Mexico) had recognition close to that of CA (~12%) with INS antivenom. In the intermediate molecular weights, INS antivenom recognized 52% of components for CA, which was also well recognized by Bioclon antivenom (61%). High molecular-weight bands were better recognized for OR by INS antivenom with 46%, and 48% for Bioclon antivenom. The low and high molecular-weight components were better recognized for *C. simus* by the INS antivenom, showing 11 and 51%, respectively.

Recognition of *C. simus* with the Mexican antivenom was lower, compared to the three Colombian venoms. Thus, results showed that there was evident recognition of the bands related to proteases and medium molecular weight components such as PLA_2_ and CTL, but not for small components. None of the antivenoms seemed to recognize crotamine. Antivipmyn-Tri^®^ already had shown this unrecognition against crotamine from the venom of the Mexican snake *Crotalus molossus nigrescens* [88] and in a recent study with INS antivenom, such doubt was also unresolved [32].

It was observed that INS antivenom also recognized, and importantly, components in a wide range for *C. simus* venom. It is possible that this cross recognition is because both lineages (*C. simus* and *C. durissus*) belong to the *C. durissus* (south) clade of the *C. durissus* complex [5], which would make them share epitopes in the components of their venoms.

The recognition of proteases between 20 and 25 kDa, and around 50 kDa, for the four venoms, by Antivipmyn-Tri^®^, is related to clinical trials considering that it counteracts hemorrhagic, local and systemic signs, in the first 6 h, and normalizes the coagulation in the first 24 h; symptoms were generated mainly by the effect of the aforementioned proteases [22,71].

The findings for INS antivenom on Colombian species are related to recent studies in which good recognition occurs towards major components and of toxicological interest in the venom of *C. d. cumanensis* [32]. INS antivenom insert indicates its use to control the appearance of neurological signs such as myasthenia, altered visual acuity, palpebral ptosis, diplopia, loss of balance and dyspnea, in addition to clinical signs that suggest myotoxicity and hemotoxicity such as myalgias, altered blood coagulation and kidney failure [21], signs that are related to the toxins that the antivenom is apparently recognizing. Although polyspecific antivenoms can recognize the most relevant toxic components of the venoms for which they have been designed [89], they also present immunorecognition by cross-reaction with other venoms. In the Colombian clinical scenario, crotalic accident requires for its treatment, half the number of ampoules of INS antivenom than those of Antivipmyn-Tri^®^ [90,91].

Antivenom of the Instituto Clodomiro Picado (ICP) of Costa Rica, of complete IgG, produced with the venoms of *B. asper*, *L. stenophrys* and *C. simus* [92] had previously been tested against the venom of *C. d. cumanensis* from Venezuela, using antivenomic techniques, showing null affinity for DIS, crotamine, SVSP, crotoxin and some PLA_2_. Likewise, the efficacy of the experimental antibotropic and anticrotalic antivenom (ABC), of equine origin, produced with the venoms of *B. colombiensis* and *C. d. cumanensis* at the Centro de Biotecnología of the Universidad Central de Venezuela, F(ab’)_2_ type, presented a similar behavior to that of ICP, except for a slightly higher recognition to crotamine and crotoxin [12]. In response to snake venom, ICP antivenom generally has better recognition of SVMP-III, LAAO, and CTL; for the other components, immunorecognition is variable or minimal, as in the case of vasoactive peptides [64].

In the case of affinity chromatography, the matrices were coupled with 39.9 mg of INS antivenom, which was close to 100% of coupling. After the venoms were added, a retention of 34%, 57% and 43% was identified for MM, CA and OR, respectively. The immunospecificity control did not have an important recognition of the venom, with 95% of the total protein not retained.

The non-retained and retained fractions were submitted on the RP-HPLC run. For analysis purposes, the three chromatograms were matched at 27 retention times. The recognition for the fractions eluted in the first 42 min (up to fraction 6) was better for CA (20%). The recognition of 11% for fraction 5, which corresponds to crotamine, stands out. For the intermediate fractions, between 11 and 14, which resulted around minute 56, recognition was 6% for MM, 3% for CA and 16% for OR. The subunit B of crotoxin appears in this strip.

**Figure 7:**
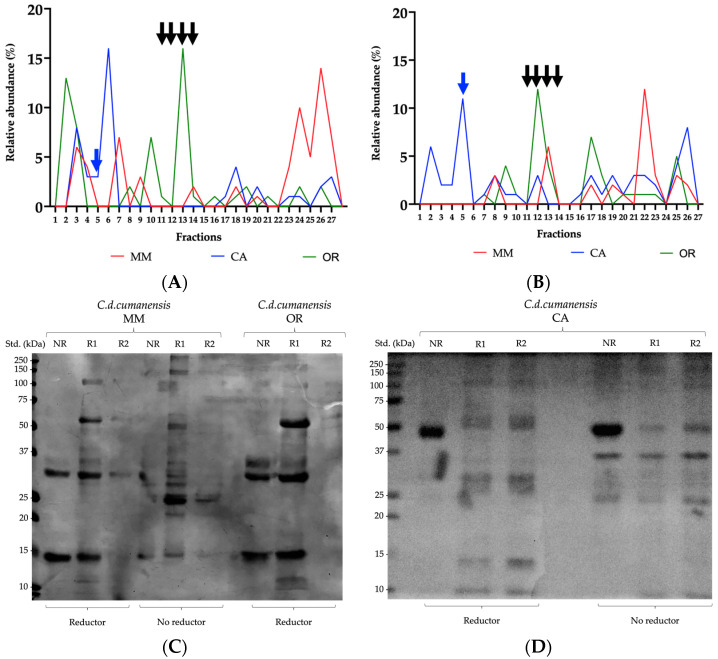
Immunorecognition of venoms by the antivenom of Instituto Nacional de Salud. Each panel shows the relative abundance obtained by RP-HPLC of what was recovered from the affinity matrix. In panel (**A**) non-retained (NR) and in (**B**) retained (R) fractions for the venom of *C. d. cumanensis* from Magdalena Medio (MM), Caribe (CA) and Orinoquía (OR). Arrows show the two neurotoxic components of importance. Blue arrow indicates the fraction corresponding to crotamine, which exceeds 10% recognition for the CA venom, and the black arrows correspond to the fractions related to PLA_2_, where the subunit B of crotoxin also appears, whose recognition is better for the OR venom, also above 10%. Panels (**C**,**D**) show the 12.5% SDS-PAGE under reducing and non-reducing conditions of the fractions recovered from the affinity matrices of each venom. An amount of 5 μg of protein per sample were seeded. Std.: molecular weight standard.

Retention times between 63 and 79 min presented with an average recognition of 17%. For the last fractions, MM and OR had retentions of 7.5% while CA reached recognitions of 14%. Subunit B of crotoxin (fraction 12) is best recognized for OR venom (12%), followed by MM (6%) and CA (3%) (see Figure 7A,B).

Fractions resulting from affinity chromatography were concentrated by ultrafiltration (3 kDa, MW cut off) to seed on electrophoresis gels, to visualize the recognized and unrecognized elements of each venom (Figure 7C,D). Bands between 50 and 70 kDa, related to oxidases [33,70,93]; bands between 25 and 100 kDa, corresponded to proteases [34,69,94]; those around 15 kDa, were compatible with PLA_2_ [36,37] and CTL [52,53]; others below 15 kDa related to DIS [55,56], and; myotoxins [57,58], show a good recognition by INS antivenom, for the three venoms, since the bands are well defined towards those zones in the lanes indicating the retained fractions. Even crotamine, for CA venom, seems to have good recognition. For MM and OR venoms, the 31, 29 and 14 kDa bands apparently demonstrate intermediate recognition. The band at 49 kDa is not recognized at all in the case of CA venom. Bands related to proteases and PLA_2_ appear in both lanes, non-retained and retained fractions, which indicates the partial immunorecognition of the antivenom.

Four large groups of protein components can be identified from what is eluted by RP-HPLC (as mentioned above), given by the percentage of solution B, as follows: Group (1) peptides and nucleosides elute from 5 to 10% of B (up to 30.6 min., fraction 1); Group (2) small proteins elute from 10 to 20% of B (up to 36.3 min, fractions 2 to 5); Group (3) medium-sized proteins elute from 20 to 30% of B (up to 60 min, fractions 6 to 14), and; Group (4) large or more hydrophobic proteins elute from 30 to 70% of B (fractions 15 to 27) [63,64,95].

**Figure 8:**
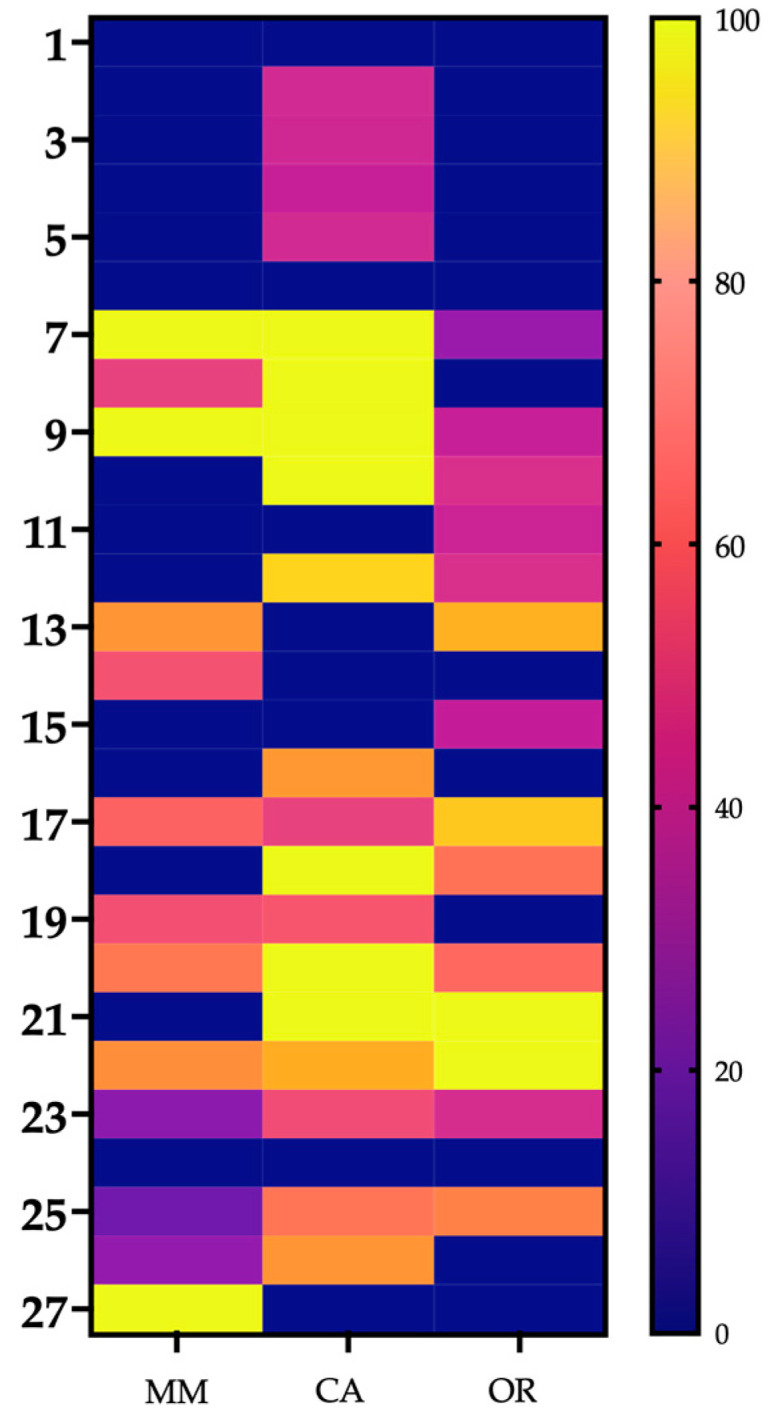
Heat map indicating immunorecognition by the Instituto Nacional de Salud antivenom for *C. d. cumanensis* of Magdalena Medio (MM), Caribe (CA) and Orinoquía (OR) venoms.

Figure 8 shows the immunorecognition of INS antivenom to the groups and fractions (left “*y*”axis) obtained by RP-HPLC of each venom (“*x*” axis). The sum of the areas under the curve of the non-retained fractions plus that of the retained fractions represents the 100% of each group (right “*y*”axis). In general, the best recognition was obtained for proteins eluted in intermediate and later fractions. The recognition of low molecular-weight proteins in CA venom is highlighted.

A second-generation antivenomics approach was used for affinity chromatography using the INS antivenom [96]. It was observed that the complete immunoglobulins G seem to have a good coupling with the Sepharose 4B matrix, since not only did the antivenom have a high percentage of union, but so did the nonspecific IgG that were used for the control of specificity. The non-retained and retained fractions of all the venoms evaluated by electrophoresis showed good recognition by well-marked bands in all ranges. Of the unrecognized fractions, some bands related to PLA_2,_ and proteases stand out. The same bands have their counterpart in the lanes for retained fractions, so a partial recognition is suggested. No prior purification of IgG from INS antivenom was undertaken. Considering that the antivenom contains other globulins, this could justify the remarkable coupling percentages achieved, which, in turn, caused steric hindrance in the affinity matrix, preventing adequate retention of some venom components, mainly those of higher molecular weight.

Some studies in the literature had described SVMP-I and DIS as poor immunogens, as suggested by some of the results obtained in the present study [64]. The fractions that showed high recognition seem to be represented by components that eluted in the range of medium molecular-weight proteins, which might be CRISP, SVSP, PLA_2_ or CTL (mainly in CA venom).

ICP antivenom in other studies had already been tested against the venom of *C. d cumanensis* from Venezuela, showing variable recognition for DIS, while poor recognition for vasoactive peptides, crotamine and crotoxin [64]. Immunodepletion analysis of a Colombian *C. d cumanensis* venom pool, with Antivipmyn-Tri^®^, showed a significant reduction for the fractions with DIS, those with the crotoxin complex, and for those with high protein content molecular mass, but not so for crotamine [31].

Recognition of OR venom by the matrix used as immunoaffinity control did not reach 5% of the protein components of the venom. This demonstrates low nonspecific binding, as has already been pointed out in other studies [96].

In this comparative study of the components of rattlesnake venom, considering the parameters of lethality, hemostatic affectation and composition, the presence of geographic differences within the species was demonstrated to have an impact on the activity of these venoms and on the response to antivenoms, which is important in the treatment of snakebite accidents.

Variation of venoms has diverse causalities [18]. Snake venoms show wide variation in composition and biological activities among species, as well as at higher taxonomic levels. This variation is interpreted as adaptative [97] because it allows prey to be available, or deters predators. Venom variability has important implications both for venom research itself and for the clinical treatment of the accident, including antivenom choice and the selection of specimens for antivenom production [97,98,99].

Intraspecific venom variability exists among populations, i.e., regional variation, as well as between ages and sizes. Venom is an ecological trait that evolves dynamically, so the variety in the snake’s diet makes a difference in the composition of its venom, anticipating that the diet also varies with the age of the specimen. Regional variation may be related to ecological variation between populations and to neutral evolution, and they function in association with positive selection. Other possible causes of venom variation are at the transcriptomic level where RNA interference regulates protein translation. In other cases, as in *C. durissus*, the duplication of crotamine genes makes the percentages in the venoms higher [67,100]. This means that for a trait to evolve rapidly, there must be considerable heritable diversity within populations, and this would support the hypothesis that variation would occur among venoms in adult members of the same population [97]. Geographic distribution as a related factor in venom variation reflects natural selection to feed on local prey [101]. The increasing frequency of crotamine in populations of the *Crotalus* genus should be a warning about the need to develop an antivenom capable of neutralizing this toxin [102].

Venoms were provided by INS, whose activity is that of a pharmaceutical laboratory that complies with the WHO guidelines to produce antivenoms through the request of individuals and the formation of representative pools of each ecoregion. It is only possible to know that these are individuals that were in captivity in the serpentarium, whose venoms conserve toxic activity, and are evaluated periodically. There is a lack of data on variables such as the number of individuals used, stage of life and sex, among others. The expansion of a study that considers these factors can complement the present one.

The subsequent complementary investigations could continue along the line of study of the evaluation of the dynamism of the venom evolution from the molecular level with proteomic and transcriptomic analyzes as a guide, the experimental modification of antivenoms or their production process, as well as the search, purification, and study of molecules for biotechnological purposes.

## 3. Conclusions

High molecular-weight components are represented by CA venom, which gave it better protease activity; those of intermediate and low molecular weight by MM venom, which provided better PLA_2_ activity, procoagulant effect and lethality, and the peptides for OR venom, which are possibly related to its better cytotoxic activity on one of the tumor cell lines tested. As a special section within the peptide component, crotamine was exclusive to CA venom.

There is a relationship between the amount of PLA_2_ within the MM and CA venoms, with their PLA_2_ activity and their cytotoxic effect on one of the study tumor cell lines. By evaluating the toxic effects in vivo, in contrast to the composition of the venom, we can conclude that MM venom is the most toxic, followed by CA and lastly OR.

Although INS antivenom has good recognition over the whole range of molecular weights for the three venoms, it seems to be better recognized in certain groups of molecular weights, per venom, which makes it peremptory to use a representative mixture of each one of them, and even consider enriching them with important components such as low molecular-weight myotoxins, which apparently do not reach a full recognition.

The study of cross-reaction recognition of other *Crotalus* venoms by the INS antivenom should be expanded, since promising results against foreign species were evident.

Explicit differences were found among the three venoms regarding the behavior of their biological activity and biochemical composition, which agrees with intraspecific variation due to geographic implications. This study used both a qualitative approach and a quantitative approach to protein characterization to answer questions about phenotypic variation in a particular adaptative trait such as venom. Likewise, the importance of variability traces in response to antivenoms made with native and foreign species lays the foundations for the design of strategies in the development of antivenoms.

## 4. Materials and Methods

### 4.1. Venoms and Antivenoms

Freeze-dried venoms of *C. d. cumanensis* from the Magdalena Medio, Caribe and Orinoquía regions, as well as *Bothrops asper* venom were provided by the Instituto Nacional de Salud (INS). Venoms of *C. d. terrificus* (Lot No. Cdt_MTox-062618, The National Natural Toxins Research Center 975 W. Avenue B, MSC 224, Kingsville, TX, USA), *Bothrops ammodytoides*, and *C. simus* were provided by the Instituto de Biotecnología of Universidad Autónoma de México. Crotamine and crotoxin subunit B were purified by RP-HPLC as described [62]. Venoms were stored at −20 °C until use [103]. It was used INS commercial antivenom, from IgG of equine origin, in liquid presentation, storage at 5 °C, against *Bothrops* spp. and *C. d. cumanensis* (Lot No. 18SAPO2, expiration October/2021); and commercial antivenom Antivipmyn-Tri^®^, type F(ab’)2 of equine origin, lyophilized, produced from *Bothrops* spp., *Lachesis* spp., and *Crotalus* spp. South American (Lot No. B-8K-31, expiration September/2022).

### 4.2. Animals

For the in vivo tests, ICR (CD-1) mice, of indistinct sex, with body weights between 16 and 20 g were used. During the experimental tests, the animals were not subjected to any additional stress than the inoculation of the venoms; the housing conditions of the animals were controlled, and they were maintained with food and water *ad libitum*. Tests were carried out in the animal facility of INS and IBt.

### 4.3. Protein Quantification

An amount of 10 mg of each venom was dissolved in 1 mL of phosphate buffered saline (PBS), and protein concentration was determined by the bicinchoninic acid (BCA) method using bovine serum albumin (BSA) as standard (Pierce™ BCA Protein Assay Kit, Thermo Scientific^TM^). The fractions obtained from RP-HPLC were quantified by Thermo Scientific™ NanoDrop 2000. The fractions obtained from the affinity column (Sepharose 4B coupled to immunoglobulins G) and the antivenoms were quantified by spectrophotometric method with absorbance at 280 nm, where one absorbance unit is equal to 1 mg/mL. For antivenoms a molar extinction coefficient of 1.4 was used, using a 1 cm light path cell (A_280_).

### 4.4. Polyacrylamide Gel Electrophoresis (SDS-PAGE)

This procedure was performed according to standard methods [104,105]. Venom samples were loaded onto 12.5% and 15% polyacrylamide gels. Precision Plus Protein^TM^ Dual Xtra Standards, Bio-Rad (ranges 10–250 kDa) and pre-stained Page Ruler^TM^ Thermo Scientific^TM^ (ranges 10–170 kDa) molecular-weight standards were used, while 2-mercaptoethanol was used for reducing conditions. Gels were stained with Coomassie R-250 and analyzed using Image Lab 5.2.1 open program (Bio-Rad Laboratories, Inc., Berkeley, CA, USA).

### 4.5. Reverse Phase High-Performance Liquid Chromatography (RP-HPLC)

The methods described in [63] were followed. Venoms were dissolved in 1 mL of water containing trifluoroacetic acid (TFA) 0.1% (solution A) and then subjected to RP-HPLC (Agilent 1100 series) using Supelco Discovery^®^ C18 analytical column (25 cm × 4.6 mm, particle size 5 µm, 595 North Harrison Road, Bellefonte, PA, USA. Cat No. 504971). Venoms were eluted with a linear gradient of acetonitrile with TFA 0.1% (solution B) as follows: 0% over 15 min, 0–15% over 15 min, 15–45% over 60 min, 45– 70% for 10 min, 70% for 9 min, 70–100% for 5 min. and finally 100% for 5 min. plus. It was monitored at a wavelength of 215 nm. The collected fractions were dried in a vacuum concentrator and stored at 4 °C.

### 4.6. Biological Activities

#### 4.6.1. Median Lethal Dose (LD_50_)

Serial dilutions of the venoms dissolved in saline solution were prepared and 500 µL were then inoculated intraperitoneally into mice (*n* = 5 per dose). Between 7 and 9 dilutions were evaluated for each venom with a dilution factor between 1.5 and 1.7 in the concentration range from 0.04 to 3.79 µg/g mouse. As a negative control, saline solution was used. The test was read 48 h later. The LD_50_ and the respective 95% confidence limits were established using the Spearman-Kärber method [19,106,107,108].

#### 4.6.2. Minimum Defibrinating Dose (MDD)

Venom was prepared at concentrations from 2.5 to 19 μg/mL in saline solution, to inject 200 μL of each solution in the caudal coccygeal vein, in groups of 5 mice. As a negative control, saline solution was used. One hour later, the mice were anesthetized by intramuscular injection of ketamine (1.5 mg) and xylazine (1 mg). By cardiac puncture, 200 μL of blood were obtained for incubation at room temperature. After 2 h, clot formation was observed. The MDD corresponds to the dose of venom that induces incoagulability. Test results were determined by Spearman-Kärber statistical analysis. [19,106,107,108].

#### 4.6.3. Minimum Coagulant Dose (MCD)

It was determined by adding a volume of 100 μL with serial dilutions of venom between 0.30 and 13.01 μg, to 200 μL aliquots of citrated human plasma, mixing gently by inversion and measuring the time of solid clot appearance in 60 s. As negative control saline solution was used and *Bothrops asper* venom was used as a positive control [19,106,107,108]. Units were converted for literature contrast purposes. Assays were performed in triplicate.

#### 4.6.4. Cytotoxicity

It was evaluated on the cell line MDA-MB-468 (ATCC^®^ HTB-132™) and MCF-7 (ATCC^®^ HTB-22™), both from ductal invasive breast adenocarcinoma. The cells were seeded in a 96-well plate at a density of 10,000 cells/well in 100 μL of RPMI culture medium; to observe dose-response effects on cell viability, they were treated for 36 h at 37 °C, 5% CO_2_ and 95% humidity with each of the venoms in a concentration range from 1 to 100 μg/mL. The effect on cellular metabolic activity was determined by colorimetric assay with MTT. At 24 and 36 h, respectively, 10 μL of MTT (5 mg/mL in PBS) were added to each of the wells and incubated for 3 h. Subsequently, the culture medium was discarded, and the formazan crystals were allowed to decant, which were solubilized with 100 μL 100% DMSO for 30 min at 37 °C. Once the solubilization process was finished, absorbance was read at 540 nm [109]. Each test was performed in triplicate and the results were evaluated using one-way ANOVA and Tukey’s multiple comparisons test.

### 4.7. Enzimatic Activities

#### 4.7.1. Phospholipase A_2_ Activity

For qualitative testing, a 10% (*w*/*v*) chicken egg yolk solution was prepared in 0.1 M Tris-HCl pH 7.5; 5 mM CaCl_2_ and 0.5% Triton X-100 mixed for 10 min and then centrifuged at 1500 rpm for 5 min. An amount of 0.2 g of agarose was dissolved in 10 mL of 0.2 M Tris-HCl pH 7.95, and 1 mL of 20 mM CaCl_2_, 7 mL of 0.1% rhodamine 6 G, 100 µL of Triton X-100, with 2 mL of 10% egg yolk solution (supernatant), to solidify in Petri dish. Circular wells were made to seed 5 µg of each sample. As a positive control, 5 µg of *Bothrops ammodytoides* venom was used, and milliQ water was used as a negative control. After incubating 1 h at 37 °C, the activity was visualized in a UV transilluminator.

The quantitative testing was determined by colorimetric method [110,111]. For the substrate, 0.6 g of lecithin were dissolved in 1 mL of ethanol at 45 °C, followed by 0.86 mL of Triton X-100, 8 mL of 0.1 M NaCl, 4 mL of phenol red 5.5 mM, 1.6 mL of 1 M CaCl_2_ and completing 16.6 mL with distilled water. The pH of the solution was adjusted by adding a drop of 2 M NaOH. Different concentrations of venom were seeded in a 96-well plate in a range between 0 and 0.66 μg/μL, adding 100 μL of substrate to each well. Incubation was carried out for 15 min at 37 °C. Deionized water was used as a negative control. It was read at a wavelength of 540 nm in a Bio-Rad model 550 microplate spectrophotometer.

#### 4.7.2. Hyaluronidase Activity

Amounts of 30 μg of each sample were loaded onto a 10% SDS-PAGE gel, copolymerized with 0.5 mg/mL *Streptococcus equi* hyaluronic acid sodium salt (Sigma^®^, St. Louis, MI, USA). After electrophoretic separation, the gels were incubated with sodium phosphate buffer pH 5.8 (0.1 M phosphates, 0.15 M NaCl, 5% Triton X-100) for 1 h at room temperature, twice. It was incubated with sodium phosphate buffer pH 5.8 (0.1 M phosphates, 0.15 M NaCl, 0.05% Triton X-100) for 1 h and finally with sodium phosphate buffer pH 5.8 (0.1 M phosphates, 0.15 NaCl) for 10 min. The gels were left in a humid chamber overnight. Subsequently, two washes were carried out with Tris-HCl pH 7.95 (0.015 M) and they were stained for 5 h under agitation, using a solution of 5% formamide, 20% isopropanol, Tris-HCl pH 7.95 (0.015 M) and 5 mL of 0.1% stains all, protected from exposure to light. Gels were distained for 1 h in agitation with 5% formamide solution, 20% isopropanol and Tris-HCl pH 7.95 (0.015 M) [112].

#### 4.7.3. Protease Activity

A 1.5 mg/mL gelatin gel and a 0.8% casein gel were copolymerized independently with 12.5% polyacrylamide. 5 μg of each venom were seeded and *Bothrops ammodytoides* venom was used as a positive control. After electrophoretic separation, the gels were incubated in 0.1 M Tris-HCl pH 8.0, 5% Triton X-100, with shaking for 1 h. They were then incubated with 0.1 M Tris-HCl pH 8.0; 0.05% Triton X-100 for another hour. The gels remained for 1 h additional in 0.1 M Tris-HCl pH 8.0. Finally, they were placed in a humid chamber overnight and stained with Coomassie R-250 blue. Gels were distained with 10% acetic acid and 10% isopropanol [113].

### 4.8. Antivenom Evaluation

#### 4.8.1. Determination of Median Effective Dose (ED_50_)

Solutions containing different concentrations of INS antivenoms were mixed with 5LD_50_/mice of venom from each region (as obtained in the lethality assays), preincubated at 37 °C for 30 min and then injected intraperitoneally in mice (*n* = 5 per dose, 200 μL/mice). Five to six different dilutions of the antivenom were tested, with dilution factors ranging from 1.2 to 1.4 and attaining concentrations of 0.3 to 32.9 mg/mL. Three control groups were used, two negative (one with antivenom and one with saline solution, 200 μL/mice) and one positive (5LD_50_ of venom/mice). The death ratio was recorded after 48 h and experiments were only considered valid when attaining death ratios of both, zero and 100%. The ED_50_ was established using the Spearman–Kärber method and expressed in milligrams (mg) of venom per milliliter (mL) of antivenom [19,107,108,114].

#### 4.8.2. Affinity Chromatography

A second-generation antivenomic approach was used [96]. The coupling was made as described by [115] with some modifications. 0.5 g of Cyanogen bromide-activated-Sepharose^®^ 4B, lyophilized powder (C9142 Sigma-Aldrich, CAS number 68987-32-6) were packed with 3 mL of prewash buffer (HCl 1 mM) under stirring for 15 min. at room temperature. It was washed with 1 mM HCl and a coupling buffer (0.2 M NaHCO_3_, 0.5 M NaCl pH 8.3) was added until the pH of the wash was >8.5. INS antivenom (40 mg) were added in a 1:1 (*v*/*v*) ratio with the resin, previously dialyzed against coupling buffer. It was left stirring overnight at 4 °C. The amount that was not retained was collected for quantification. The column was washed with a coupling buffer. The blocking buffer (0.1 M Tris-HCl pH 8.0) was passed and left stirring at room temperature for 4 h. To remove antibodies not bound to the column, six interspersed washes were performed with wash buffer (0.1 M acetic acid/sodium acetate, 0.5 M NaCl) and blocking buffer, neutralizing pH at the end with PBS. For specificity control, the same procedure was carried out, coupling IgG from preimmunized horses.

An amount of 300 μg of protein of each venom in 400 μL of PBS were passed through the matrices and left under rotary agitation at room temperature for 1 h. The non-retained fraction was eluted with PBS, the retained fraction 1 with 0.1 M acetic acid pH 2.4, neutralizing with 1 M Tris-HCl buffer pH 9.0; and retentate 2 with 50 mM sodium hydroxide, neutralizing with 0.1 M acetic acid pH 2.4. Each fraction was centrifuged at 13,000 rpm for 2 min to use the supernatant and concentrate by Amicon^®^ (3 kDa, MW cutoff).

#### 4.8.3. Western Blotting

Amounts of 5 µg of total protein of each venom were seeded in 15% SDS-PAGE under non-reducing conditions, which were subsequently transferred to a nitrocellulose membrane for 1 h with constant current of 400 mA in a semi-humid chamber (OWL). The membrane was blocked at 4 °C overnight in blocking buffer (5% skim milk powder, in PBS/0.5% Tween 20; 1X TBST). It was washed three times with 1X TBST and incubated with the primary antibody (INS antivenom or Antivipmyn-Tri^®^) at a concentration of 200 µg/mL in TBST in a final volume of 10 mL, under rotating agitation for 1 h at room temperature. It was washed three times with TBST 1X and incubated with the secondary antibody (0.5 mg Affinity purified antibody peroxidase labeled goat anti-horse IgG (H + L). Lot. No. 120607, KPL. 910 Clopper Road, Gaithersburg, MD 20878 USA), prepared at a dilution of 1:7000 in TBST 1X, and it was left under rotary agitation for 1 h. at room temperature. Finally, it was washed three times with TBST 1X and 1 mL of TMB blotting solution was added to reveal (Thermo Scientific, Rockford, IL, USA) [116].

#### 4.8.4. Enzyme-Linked Immunosorbent Assay—ELISA

The samples were prepared in a sensitization buffer (100 mM carbonate/bicarbonate pH 9.5) at a concentration of 4 μg/mL and 100 μL were seeded in each well, in duplicate. It was incubated at 37 °C for 1 h. Subsequently, the content was discarded, and each well was washed with 200 μL of washing buffer (Tris-HCl 50 mM pH 8.0, NaCl 150 mM), three times. 200 μL of blocking buffer (50 mM Tris-HCl pH 8.0, 5 mg/mL gelatin, 0.02% Tween 20) were placed and left at 4 °C overnight. INS antivenom was prepared in 50 mM Tris-HCl buffer pH 8.0, 0.5 M NaCl, 1 mg/mL gelatin, 0.05% Tween 20) at a concentration of 700 µg/mL and seeded 100 μL, making serial 1:3 dilutions with vehicle buffer. Each well was previously washed with 200 μL of wash buffer, three times. It was left in an incubator at 37 °C for 1 h. Each well was washed with 200 μL of washing buffer, three times, and 100 μL were placed in each well with the preparation of the secondary antibody (Affinity purified antibody peroxidase labeled goat anti-horse IgG (H + L) of 0.5 mg), prepared in vehicle buffer at a 1:4000 dilution. It was left in an incubator at 37 °C for 1 h. It was revealed with ABTS in 70 mM Citrate-Phosphate buffer pH 4.2 and 0.02 µL of H_2_O_2_. The reading was made in a spectrophotometer at 405 nm at 60 min.

### 4.9. Statistics

For ELISA and cytotoxicity assays, slope variable nonlinear regression analyzes were performed. For all statistical analyses, as well as for the determination of mean values, standard deviations, coefficients of variation, and 95% confidence intervals, Prism 9.0 software (GraphPad, San Diego, CA, USA) was used. One-step ANOVA test, multiple comparisons test and Tukey’s test were used in the analysis of biological tests (significant *p* value < 0.05).

## Figures and Tables

**Table 1:** Protein families in *Crotalus durissus cumanensis* venoms obtained by SDS-PAGE and RP-HPLC grouped by molecular weight.

SDS-PAGE				RP-HPLC # Fraction	Possible Related Protein Families	Reference
Molecular Weight (kDa)	Relative Intensity (%) by Ecoregion					
	MM	CA	OR			
113.1	11.4	9.5	5.0	11–14	LAAO, SVMP, HYA	[33,42,43,44,45,46,47]
83.7	8.8	6.7	3.5			
73.2	9.9	21.2	8.3			
64.0	9.0	6.9	0.0			
57.3	4.5	0.0	0.0	9–12	SVMP, SVSP, CRISP	[31,33,34,42,43,44,48,49,50,51]
54.5	0.0	4.4	0.0			
51.4	3.6	0.0	0.0			
46.2	4.8	7.0	4.1			
41.1	5.0	0.0	0.0			
37.2	11.8	7.6	6.6			
32.7	5.8	3.1	2.9			
29.9	5.1	5.5	2.6			
25.7	6.0	9.6	0.0			
13.0	30.1	28.0	13.1	5–8	PLA_2_, CTL, CRISP, growth factors	[35,36,37,51,52,53,54]
11.4	9.3	14.7	4.9			
9.8	13.5	0.0	0.0	1–6	DIS, low molecular weight myotoxins, vasoactive peptides	[55,56,57,58,59,60]
8.9	16.4	19.4	0.0			
7.8	15.1	35.6	0.0			

LAAO: L-amino acid oxidases; SVMP: metalloproteases; HYA: hyaluronidases; SVSP: serine proteases; CRISP: cysteine-rich secretory proteins; PLA_2_: phospholipases A_2_; CTL: C-type lectins; DIS: disintegrins; MM: Magdalena Medio; CA: Caribe and OR: Orinoquía. Relative intensities represent a single measurement on a gel.

**Table 2:** Identification of the abundant fractions obtained by RP-HPLC for *Crotalus durissus cumanensis* venoms.

Fraction	RT	RA MM (%)	Molecular Mass (Da)	RA CA (%)	Molecular Mass (Da)	RA OR (%)	Molecular Mass (Da)
1	31.0	2	ND	3	ND	2	ND
2	36.6	1	ND	10	4910.9	2	ND
3	39.9	6	1239.1	1	ND	6	ND
4	45.3	2	ND	2	ND	6	ND
5	47.5	18	ND	6	ND	15	ND
6	49.1	5	ND	3	ND	5	ND
7	55.3	18	14,395.6	19	ND	16	ND
8	64.1	3	ND	1	ND	1	13,550.0
9	66.2	14	15,424.5	10	ND	10	ND
10	69.9	4	ND	1	ND	6	ND
11	73.4	4	14,439.0	1	ND	3	ND
12	75.8	6	ND	8	ND	4	ND
13	77.6	9	ND	12	ND	11	ND
14	81.5	6	ND	24	ND	13	ND

RT: Retention time; RA: Relative abundance; MM: Magdalena Medio; CA: Caribe and OR: Orinoquía; ND: not determined. Relative abundances represent a single measurement on a gel.

**Table 3:** Relationships among phospholipase A_2_ (PLA_2_) relative abundances and whole *C. d. cumanensis* venom cytotoxicity on the cancer cell lines MCF-7 and HTB-132. IC_50_: concentration at which 50% of cells survive. It was determined using the Prism 9.0 program.

Venom	IC_50_ MCF-7 (μg/mL)	IC_50_ HTB-132 (μg/mL)	PLA_2_ (%) ^1^	PLA_2_ (μg/mL) vs. IC_50_ MCF-7 ^2^	PLA_2_ (μg/mL) vs. IC_50_ HTB-132 ^2^
Magdalena Medio (MM)	0.4	9.3	44	7.7	8.3
Caribe (CA)	0.9	3.7	32	5.0	4.2
Orinoquía (OR)	0.7	3.5	36	8.0	7.6

^1^ Added percentages of fractions 7–11 obtained by RP-HPLC [63]. ^2^ Intercept of trend lines for cytotoxicity and PLA_2_ concentration. It is defined as the concentration of PLA_2_ necessary to reach the IC_50_ in each cell line.

**Table 4:** Individual variation of toxic activities for *Crotalus durissus cumanensis* venoms from three Colombian regions.

Ecoregion	Median Lethal Dose (LD_50,_ µg/g ^1^)	Minimum Defibrinating Dose (MDD, µg/g ^1^)	Minimum Coagulant Dose (MCD, mg/L)
Magdalena Medio (MM)	0.07 ± 0.009	0.08 ± 0.007	40.5 ± 2.8 ^4^
Caribe (CA)	0.10 ^2,3^ ± 0.005	0.10 ± 0.007	66.7 ± 2.6
Orinoquía (OR)	0.08 ± 0.007	0.11 ± 0.018	ND

Data is defined by the average of three measurements and its standard deviation is indicated. ^1^ Average mouse weight used for calculation: 18 g. ^2^ MM y CA *p* value < 0.05. ^3^ OR y CA *p* value < 0.05. ^4^ MM y CA *p* value < 0.05.

## Data Availability

Not applicable.
